# Beneath the Surface: The Emerging Role of Ultra-Processed Foods in Obesity-Related Cancer

**DOI:** 10.1007/s11912-025-01654-6

**Published:** 2025-02-27

**Authors:** Ioanna A. Anastasiou, Dimitris Kounatidis, Natalia G. Vallianou, Alexandros Skourtis, Krystalia Dimitriou, Ilektra Tzivaki, Georgios Tsioulos, Anastasia Rigatou, Irene Karampela, Maria Dalamaga

**Affiliations:** 1 Diabetes CenterDepartment of Propaedeutic Internal MedicineMedical School, Laiko General Hospital, National and Kapodistrian University of Athens, FirstAthens, Greece; 2 Department of Pharmacology, National and Kapodistrian University of Athens, 11527 Athens, Greece; 3 First Department of Internal Medicine, Sismanogleio General Hospital, 15126 Athens, Greece; 4 Department of Internal Medicine, Evangelismos General Hospital, 10676 Athens, Greece; 5 Second Department of Internal Medicine, Medical School, National &, Hippokratio General Hospital, Kapodistrian University of Athens, 11527 Athens, Greece; 6 Fourth Department of Internal Medicine, Medical School, Attikon General University Hospital, National and Kapodistrian University of Athens, 12462 Athens, Greece; 7 Second Department of Critical Care, Medical School, Attikon General University Hospital, National and Kapodistrian University of Athens, Athens, Greece; 8 Department of Biological Chemistry, National and Kapodistrian University of Athens, 11527 Athens, Greece

**Keywords:** Cancer, Chronic low-grade inflammation, Gut microbiome, Obesity, Ultra-processed foods

## Abstract

**Purposeof Review:**

Ultra-processed foods (UPFs) are becoming more and more important in daily diets around the world; in some cases, they can account for as much as 60% of daily energy intake. Epidemiological evidence suggests that this shift toward high levels of food processing may be partially responsible for the global obesity epidemic and the rise in the prevalence of chronic diseases.

**Recent Findings:**

Few prospective studies have examined the relationship between UPF consumption and cancer outcomes. According to currently available information, UPFs may increase the risk of cancer due to their obesogenic properties and exposure to substances that can cause cancer, such as certain food additives and pollution from product processing. The complex relationship between obesity and cancer involves factors such as immune dysregulation, altered adipokine and sex hormone levels, abnormal fatty acid metabolism, extracellular matrix remodeling, and chronic inflammation. Addressing cancer risk associated with UPF consumption could involve a multifaceted approach, including consumer behavior modification programs and robust public health regulations aimed at enhancing food environments. Improved knowledge of the potential dual negative impacts of UPFs on the environment and cancer risk is one of the priority areas we identify for future research and policy implications. Various approaches could be used to prevent cancers associated with UPF consumption, such as consumer behavior change programs and stricter public health regulations needed to improve the food environment.

**Summary:**

This review examines for the first time the potential role of UPFs in cancer risk associated with obesity, exploring underlying biological mechanisms and identifying key areas for future research and policy action, including the dual environmental and health impact of UPFs.

## Introduction

Food processing encompasses various techniques used to transform raw foods into forms suitable for cooking, storage, or direct consumption. These processes include grinding, packaging, heating, freezing, washing, and fermenting [1]. In response to concerns over the potential adverse effects of industrial food processing on diet quality and chronic disease risk, food classification systems have been developed to categorize different types of processed foods [2]. Among these, the NOVA classification system is the most widely used; it introduced the term “ultra-processed foods” (UPFs) to describe foods subjected to the highest levels of industrial processing [3]. UPFs are often highly palatable, convenient, shelf-stable, and affordably priced, making them widely accessible and heavily marketed to consumers [4, 5].

Over the past decade, numerous meta-analyses have synthesized research examining the associations between UPF consumption and negative health outcomes [6, 7]. Increased UPF intake has emerged as a major contributor to rising global obesity rates [8], with observational studies consistently linking high UPF consumption to weight gain, overweight status, and obesity [8–11]. The World Obesity Atlas 2023 report reveals that 38% of people worldwide are classified as overweight or obese, with a body mass index (BMI) exceeding 25 kg/m^2^ [12]. In industrialized nations, 60–70% of adults have excess body weight. Obesity is a significant risk factor for numerous chronic conditions, including hypertension, dyslipidemia, metabolic syndrome, type 2 diabetes mellitus (T2DM), cardiovascular diseases (CVD), metabolic dysfunction–associated steatotic liver disease (MASLD), sleep apnea, autoimmune disorders, and various types of cancer in at least 13 different anatomical locations, including endometrial, esophageal, renal, and pancreatic adenocarcinomas, as well as hepatocellular carcinoma, gastric cardia cancer, meningioma, multiple myeloma, and cancers of the colorectum, postmenopausal breast, ovary, gallbladder, and thyroid [13–18].

Additionally, an expanding body of research suggests that UPF consumption may be associated with an increased risk of cancer and cancer-related mortality [19]. Recently, several prospective studies have begun exploring the etiological factors underlying obesity in cancer patients, aiming to clarify the links between UPFs, obesity, and cancer [20].

Till now, there are very few recent reviews, mainly systematic or umbrella, focusing on UPF and cancer risk in general or UPF and specific cancers such as gastrointestinal, breast, liver and urological cancers [21–29]. This narrative review synthesizes for the first time current evidence on the etiological links between UPF consumption and obesity-related cancer risk, integrating key epidemiological findings to clarify these interconnected health risks. Additionally, it explores potential underlying mechanisms, including the emerging role of gut dysbiosis, to provide a comprehensive understanding of how UPFs may contribute to cancer-related obesity.

### Defining Ultra-Processed Foods

The NOVA classification system suggests that excessive consumption of UPFs may pose significant health risks [30]. The detrimental effects associated with UPFs are largely attributed to their nutritional composition and processing methods [30]. UPFs are defined as industrially formulated products composed primarily of chemically altered ingredients, combined with additives to enhance flavor, texture, and appearance [4]. Analyses of global sales data and UPF consumption patterns reveal a notable shift toward a more ultra-processed diet worldwide, although this trend shows considerable variation across regions and countries [31]. For example, the percentage of dietary energy from UPFs varies substantially among high-income countries, ranging from approximately 10% in Italy and 25% in South Korea to as high as 58% in the United States and 42% in Australia [30]. In lower- and middle-income countries, such as Mexico and Colombia, UPFs constitute 16% to 30% of total energy intake, respectively [16]. Over recent decades, the diversity and accessibility of UPFs have expanded rapidly in a wide array of countries with varying economic statuses, especially in densely populated low- and middle-income nations [31–33].

Shifts in global dietary patterns from unprocessed and minimally processed foods to UPFs have been largely influenced by food environments, commercial marketing, and consumer behavior. These factors, combined with the distinctive properties of UPFs, raise concerns about overall diet quality and public health [34–36]. For instance, UPFs often contain altered food matrices, processing contaminants, and a range of industrial additives, which contribute to less favorable nutrient profiles, characterized by higher energy density, salt, sugar, and saturated fat, and lower levels of fiber, micronutrients, and essential vitamins [37]. While mechanistic research remains in its early stages, accumulating evidence suggests that these attributes may exert compounded or synergistic effects on chronic inflammatory diseases through potential physiological mechanisms, such as gut microbiome dysregulation and increased inflammation [23, 37–39].

Given these health implications, the role of UPFs in shaping dietary patterns and serving as modifiable risk factors for chronic diseases and mortality has recently become a focal point for researchers, public health advocates, and the general public.

### Ultra-Processed Foods and Obesity: Epidemiological Evidence and Potential Mechanisms

In recent years, a substantial body of research has highlighted the significant association between UPF consumption and the increasing prevalence of obesity, based primarily on observational studies [8]. While this relationship is well-documented among adults, findings in children and adolescents are less conclusive and occasionally conflicting [40]. Meta-analyses consistently demonstrate a dose–response effect, whereby higher UPF intake is associated with an elevated risk of both overweight and obesity [41, 42]. A recent meta-analysis synthesizing data from nine cross-sectional and three cohort studies quantified this association, indicating that each 10% increase in daily caloric intake from UPFs correlates with a 7% and 6% rise in the risk of overweight and obesity, respectively. Notably, this analysis also linked UPF intake to an increased likelihood of abdominal obesity [42]. Reducing UPF consumption is now seen as a promising strategy for both the prevention and management of obesity [42]. A seminal study by Hall et al. explored UPFs’ effect on caloric intake and body weight in a tightly controlled trial involving 20 weight-stable adults, revealing that participants consumed an additional 508 kcal/day on a UPF diet. This caloric increase, largely from higher carbohydrate and fat intake, led to an average weight gain of 0.9 kg, which was reversed during a phase with unprocessed foods [43].

The mechanisms underlying the link between UPF intake and weight gain are multifaceted. UPFs are often low in nutrient density yet high in energy density, which, combined with enhanced texture, taste, and the inclusion of additives that promote hyperpalatability, predisposes individuals to overconsume them. Additionally, UPFs are widely available, affordable, and frequently sold in large portion sizes, with aggressive marketing further driving consumption [44, 45].

Diets high in UPFs are linked to indicators of poor nutritional quality, including elevated levels of added sugars, saturated fats, and sodium, as well as higher energy density. These diets tend to lack essential nutrients, with lower amounts of fiber, protein, and vital micronutrients. UPFs often replace more nutrient-dense options like fruits, vegetables, legumes, nuts, and seeds, reducing the intake of beneficial bioactive compounds such as polyphenols and phytoestrogens. This nutrient-deficient dietary pattern has been associated with the prevalence and development of obesity and related conditions, primarily through inflammatory and oxidative stress pathways [23, 38, 39].

While some researchers argue that nutrient profiling alone can account for the health risks associated with UPFs, recent findings suggest that even when macronutrients are matched, UPF and minimally processed food diets have different effects on energy intake and body weight. Prospective cohort studies further support that the association between UPF consumption and obesity persists even after controlling for overall dietary quality, implying that the effects of UPFs on weight gain extend beyond their nutrient composition [43, 44, 46].

### Obesity and Cancer Risk: Pathophysiological Mechanisms and Epidemiological Insights

Obesity, defined by the World Health Organization (WHO) as a BMI over 30 kg/m^2^, has risen globally at an alarming rate, now posing a major health challenge that affects nearly 600 million adults worldwide [47]. This rapid increase is driven by diverse risk factors, including genetic predisposition and environmental influences such as aging, sedentary lifestyles, and high-calorie diets [48]. Additionally, emerging evidence highlights the role of synthetic chemicals with endocrine-disrupting properties that can alter adipocyte function, further compounding obesity risk [49]. Obesity is linked to numerous chronic conditions, including T2DM, chronic kidney disease (CKD), CVD, and mental health disorders [50–52]. Importantly, substantial evidence now connects obesity to an elevated risk of various cancers, while also adversely impacting survival rates and recurrence risks among patients with cancer [53, 54]. Consequently, obesity management has become a critical strategy for improving outcomes in patients with early-stage cancer, underscoring the need for integrated approaches to address both obesity and cancer risk.

### The Pathophysiological Link Between Obesity and Cancer Development

As previously discussed, obesity significantly heightens the risk of various cancers, with adipose tissue playing an influential role in cancer metastasis, carcinogenesis, and progression [55, 56]. The mechanisms underlying obesity-associated carcinogenesis are complex and not entirely understood. However, several biological processes are implicated, including immune dysregulation, fatty acid metabolism, extracellular matrix remodeling, hormone dysregulation, alterations in gut microbiota, and chronic inflammation. The impact of these factors may vary across different types of cancers, suggesting distinct mechanistic pathways for each cancer type [57, 58].

One primary mechanism linking obesity to cancer risk involves the function of adipose tissue as an endocrine organ. Adipose tissue, particularly due to the presence of aromatase, converts androgens to estradiol, increasing circulating estrogen levels. This rise in estrogens, predominantly in peripheral adipose tissue, has been linked to a heightened risk of endometrial, breast, and ovarian cancers [59, 60]. The second major mechanism is related to insulin resistance and hyperinsulinemia. Elevated BMI prolongs the action of insulin-like growth factor-I (IGF-1) due to sustained high levels of insulin in individuals with obesity. This condition not only precedes T2DM but is also a known risk factor for cancers, such as those of the prostate, colon, kidneys, and endometrium, driven by increased IGF-1 and insulin [61]. Insulin and IGF activate various tumor-promoting mechanisms in target cells, contributing to processes such as cell proliferation, resistance to apoptosis, angiogenesis, and lymphangiogenesis [62–64].

**Fig. 1:**
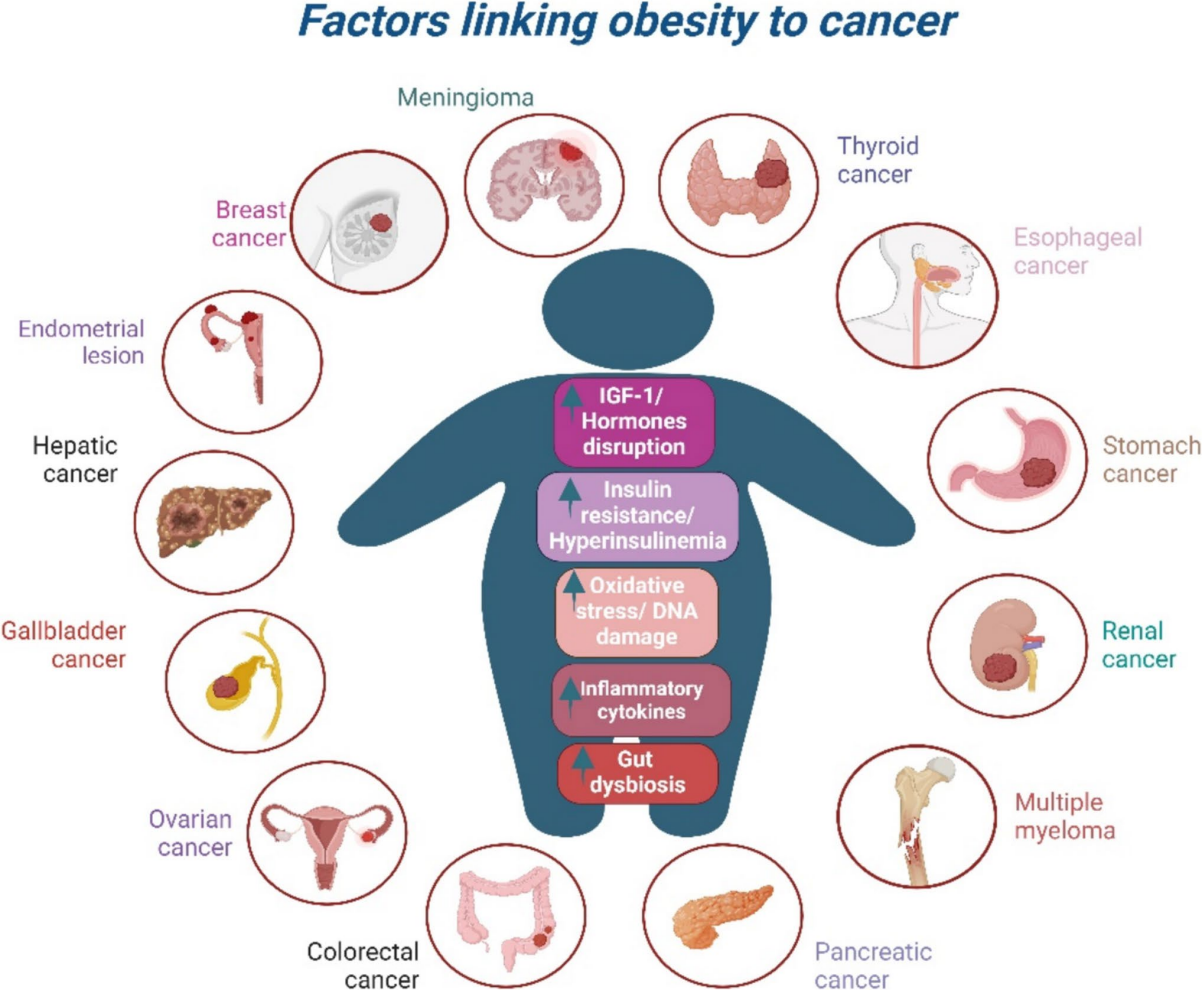
Key factors linking obesity to cancer. Abbreviations: IGF-1: Insulin-like growth factor 1; Created in BioRender. Anastasiou IA. (2025) https://BioRender.com/j66y658. Assessed on 18 January 2025

Adipose tissue also secretes adipokines, such as leptin and adiponectin, which contribute to a pro-inflammatory and pro-carcinogenic environment [65]. Obesity is associated with increased levels of leptin, which are linked to inflammation and tumorigenesis, and decreased adiponectin levels, which typically exhibit anti-proliferative properties [66]. Chronic low-grade inflammation stemming from adipocyte hypertrophy and cell death in obesity has emerged as a significant carcinogenic factor, elevating risks for cancers of the liver, biliary tract, and other organs [67]. This inflammatory state is characterized by the release of cytokines such as tumor necrosis factor-alpha (TNF-α) and interleukin-6 (IL-6), which, in turn, contribute to oxidative stress and DNA damage. Furthermore, structural changes in the tissues surrounding tumors, coupled with altered immune responses, create an environment that promotes cancer progression [67, 68]. Notably, metastasis in obesity-related cancers has been linked to various mechanisms, including angiogenesis, extracellular matrix modulation, metabolic shifts, systemic inflammation, immune cell modulation, adipokines, and extracellular vesicles like exosomes [58]. Figure 1 presents the main key factors linking obesity to cancer.

### Epidemiological Evidence on Obesity-Related Cancer Risks

Research has indicated that weight gain during adulthood is strongly associated with a higher risk for prostate, colorectal, endometrial, and post-menopausal breast cancers [69]. A significant population-based study involving over 5 million participants showed a strong correlation between increased BMI and the incidence of many common cancers [70]. Further studies utilizing Mendelian randomization techniques have demonstrated a causal relationship between higher body fat and malignancy, including esophageal, gastric, pancreatic, renal, colorectal, ovarian, and endometrial cancers [71–77].

The International Agency for Research on Cancer (IARC) in 2020 concluded a direct link between obesity and the risk of 13 different cancers, including postmenopausal breast, colorectal, endometrial, esophageal, pancreatic, renal, liver, stomach, gallbladder, ovarian, and thyroid malignancies, as well as multiple myeloma, and meningioma [78]. There is also moderate evidence for associations with other cancers, including diffuse large B-cell lymphoma, throat and laryngeal cancers, prostate cancer, male breast cancer, and oral cancer [58].

Based on an extensive analysis of 204 systematic reviews and meta-analyses investigating the relationship between excess body fat and cancer risk, men experience a 9% increased risk of developing rectal cancer (relative risk [RR] 1.09, 95% confidence interval [CI]: 1.06–1.13) and a 56% increase in risk for biliary tract cancer (RR 1.56, 95% CI: 1.34–1.81) for every 5 kg/m^2^ rise in BMI [79]. For women, each 5 kg gained in adulthood raises the risk of postmenopausal breast cancer by 11% among those not receiving hormone replacement therapy, while a 0.1 increase in waist-to-hip ratio elevates endometrial cancer risk by 21% (RR 1.21, 95% CI: 1.13–1.29). The relationship between obesity and breast cancer risk varies by menopausal status: obesity is inversely or neutrally associated with breast cancer risk in premenopausal women but positively associated in postmenopausal women, especially for hormone receptor-positive breast cancer [80–82]. Additionally, women with a normal BMI but elevated whole-body fat levels face an increased risk of breast cancer, with an adjusted hazard ratio (HR) of 1.89 (95% CI: 1.21–2.95) for all invasive breast cancer and 2.21 (95% CI: 1.23–3.67) for hormone receptor-positive breast cancer. For every 5 kg increase in trunk fat, the risk of hormone receptor-positive breast cancer rises by 56% [83].

The impact of obesity on colorectal cancer risk among individuals with Lynch Syndrome appears to vary by gender. A meta-analysis of four studies involving individuals with Lynch syndrome found that obese men had a doubled risk of colon and rectal cancer compared to men of a healthy weight (summary relative risk [SRR] = 2.09, 95% CI: 1.23–3.55). In contrast, there was no significant increase in colorectal cancer risk for females. Additionally, individuals with a germline MLH1 gene mutation had a 49% higher likelihood of developing colorectal cancer compared to those at a healthy weight (SRR = 1.49, 95% CI: 1.11–1.99) [84].

Interestingly, bariatric surgery has demonstrated a significant reduction in the incidence of various cancers, including endometrial, breast, colorectal, non-Hodgkin lymphoma, and melanoma. Overall, weight loss resulting from bariatric surgery has been associated with a 40–50% decrease in cancer-related mortality across different cancer types [85–87]. These findings emphasize the potential of weight loss as an effective preventive strategy for obesity-related cancers.

Overweight and obesity are associated with an increased risk of all-cause mortality, as shown in a systematic review and meta-analysis of 230 cohort studies [88]. Obesity is also linked to poorer outcomes for some cancers, though the relationship between obesity and cancer prognosis is less clearly defined. Differences in malignancies and even in cancer subtypes contribute to the variability in establishing a causal link. A systematic review and meta-analysis of 203 studies encompassing 6.3 million individuals with cancer revealed that obesity is associated with both higher overall and cancer-specific mortality rates. Specifically, overweight status is linked to a 17% increase in cancer-specific mortality risk (HR for cancer-specific survival, 1.17; 95% CI: 1.12–1.23; p < 0.001) and a 14% increase in overall mortality risk (HR for overall survival, 1.14; 95% CI: 1.09–1.19; p < 0.001). Additionally, obesity is associated with a 13% higher risk of cancer recurrence (HR for recurrence, 1.13; 95% CI: 1.07–1.19; p < 0.001). Among cancer types, lower overall survival rates are observed in obese patients with uterine, colorectal, or breast cancers, while obesity is linked to higher cancer-specific mortality in patients with pancreatic, breast, colorectal, prostate, and colorectal cancers, along with higher recurrence rates in these groups. Interestingly, obese patients with kidney, lung, and melanoma cancers have better survival outcomes compared to non-obese patients [41, 89].

Low physical activity, undertreatment, hormonal factors linked to certain endocrine-dependent cancers, and underlying metabolic syndrome may all contribute to the poorer outcomes observed in obese patients with specific cancers. Notably, all cancer types are impacted by dose-capping practices in patients with obesity, as discussed below [90]. Additionally, research suggests that obese cancer patients experience poorer outcomes with other conventional therapies. For example, surgical procedures in obese patients are associated with a higher risk of postoperative complications, such as wound infection, prolonged operative times, and increased blood loss [91]. Evidence indicates that patients with gastrointestinal cancers who experience complications during radical surgery are less likely to achieve favorable long-term outcomes [92]. Similarly, radiation therapy has shown reduced efficacy in obese patients, which may be attributed to daily setup challenges and the increased mobility of tumors within the patient’s adipose tissue [93].

### Cancer-Specific Analysis

#### Esophageal Adenocarcinoma

Numerous epidemiological studies have shown that obesity plays an important role in the increased incidence of esophageal adenocarcinoma [94]. Obesity-induced acid reflux disease is a significant risk factor for Barrett's esophagus, a precursor to esophageal adenocarcinoma. Chronic inflammation associated with obesity may partly explain this progression. However, obesity's link to increased risks of various cancers suggests additional contributing factors. Evidence points to a connection between body fat distribution and the risks of Barrett's esophagus and esophageal adenocarcinoma. Metabolic syndrome, characterized by altered metabolic profiles, may play a pivotal role in promoting cell cycle and genetic abnormalities that drive Barrett's progression to cancer. Research underscores the influence of metabolic syndrome on Barrett's esophagus length, systemic inflammation, and insulin resistance, highlighting its unique role in cancer risk [94].

Researchers conducted a meta-analysis to determine whether obesity is related to cancer or high-grade dysplasia in patients with Barrett's esophagus. A total of 38,565 patients (74.4% male) were the subjects of 20 studies, of which 1,684 had a high-grade dysplasia or cancer diagnosis [95]. Nineteen studies were considered moderate to high quality. Eight cohort studies reported data on 6,647 male patients with baseline nondysplastic Barrett's esophagus / grade dysplasia, of whom 555 progressed to high-grade dysplasia /esophageal adenocarcinoma (pooled annual rate of progression, 0.02%; 95% CI, 0.01%–0.03%), and 1,992 female patients with baseline nondysplastic Barrett's esophagus / grade dysplasia, with 110 progressors (pooled annual rate of progression, 0.01%; 95% CI, 0.01%–0.02%). There was no significant difference in pooled annual rate of progression between males and females (p = 0.15). Each 5-kg/m^2^ increase in BMI was associated with a 6% increase in the risk of malignant progression (adjusted OR, 1.06; 95% CI, 1.02–1.10; p < 0.001). Based on this meta-analysis, there is some evidence that obesity, as determined by BMI, is associated with a dose–response relationship with Barrett's esophageal cancer progression and malignancy [95].

### Breast Cancer

Obesity is associated with poorer survival and increased risk of recurrence among breast cancer survivors [96]. Studies reveal that post-diagnosis weight gain of over 10% increases the risk of all-cause mortality by 23%, particularly affecting those with hormone receptor-positive breast cancer [97, 98]. Obesity raises the risk of all-cause and breast cancer-specific mortality by 35% and 41%, respectively, according to a meta-analysis of 82 studies on breast cancer survivors [99]. Compared to normal-weight women, obese women had lower overall survival (HR = 1.29; 95% CI: 1.11–1.51) and disease-free survival (HR = 1.26; 95% CI: 1.09–1.46). 13 studies with 8944 women with triple-negative breast cancer were analyzed. Between 30 and 50% of women may continue to gain more than 5% of their body weight during and after chemotherapy in the first five years following diagnosis [99].

Recurrence-related post-diagnostic obesity has been demonstrated to be an unreliable diagnostic marker [100]. For instance, it has been reported that gaining 5 kg of weight within 6 months of diagnosis is a 31% worse prognostic marker (95% CI: 0.97–1.75); weight loss did not substantially follow the same pattern. A different systemic review and meta-analysis comprising 12 studies and 23,932 breast cancer survivors demonstrated that gaining weight following a breast cancer diagnosis was linked to a 13% higher risk of dying from all causes than maintaining baseline body weight [101]. With weight gain of more than 10%, there was clear evidence of an increased risk of death (HR of 1.23). Obesity has also been demonstrated to reduce the effectiveness of adjuvant aromatase inhibitors in women with hormone receptor-positive breast cancer because it increases the activity of peripheral aromatase inhibitors [98].

### Colorectal Cancer

Evidence demonstrates that a patient’s BMI significantly impacts the prognosis of colorectal cancer. Data indicate that obesity is linked to a higher incidence of advanced-stage colorectal cancer (Stages II or III) and a greater number of lymph node metastases [102]. Additionally, pre-diagnostic obesity correlates with reduced overall survival rates and an elevated risk of disease-specific mortality [103]. Specifically, obesity has been associated with a 14% increase in mortality due to colorectal cancer as well as all-cause mortality [104]. A meta-analysis of 16 prospective cohort studies involving 58,917 patients reported that a BMI ≥ 35 kg/m^2^ post-diagnosis correlates with a 13% increase in the risk of all-cause mortality. Obesity prior to diagnosis was associated with a 25% increased risk of mortality from all causes and a 22% higher likelihood of death specifically from colorectal cancer [105].

### Pancreatic Cancer

In pancreatic cancer, unfavorable outcomes have been linked to both increased fat mass and obesity, as well as reduced muscle mass and function [106]. Research by Petrelli et al. found that obesity corresponds to a 28% increase in the risk of death from pancreatic cancer [54]. Furthermore, a systematic review and meta-analysis of 13 studies determined that each incremental kilogram in BMI is associated with a 10% rise in mortality risk [107].

### Endometrial Cancer

There is a well-established association between obesity and poorer clinical outcomes in women diagnosed with endometrial cancer [108]. Obesity is also recognized as a significant risk factor for endometrial cancer itself. In terms of both disease-free and overall survival, survivors of endometrial cancer with elevated BMI and waist circumference show lower survival rates, both pre- and post-diagnosis [54, 109]. A meta-analysis encompassing 18 studies concluded that an increase of 10% in BMI is linked to a 9.2% rise in the odds of all-cause mortality, highlighting those women with endometrial cancer, especially those with a BMI of 40 or higher, face significantly elevated mortality risks [110].

### Prostate Cancer

The risk of biochemical recurrence in prostate cancer rises by approximately 21% with every 5 kg/m^2^ increase in BMI [111]. A comprehensive review and meta-analysis of 59 studies involving 280,199 patients demonstrated that obesity increases the risk of all-cause mortality by 9% and prostate cancer-specific mortality by 19%. Further, every 5 kg/m^2^ increase in BMI elevates prostate cancer mortality by 9% and overall mortality by 3% [112]. Among men with obesity diagnosed with prostate cancer, several factors are associated with adverse outcomes, including biologically aggressive cancer types, diagnostic delays, and lower success rates with treatments such as radical prostatectomy and cases with positive resection margins [113]. Table 1 summarizes meta-analysis data on the relationship between obesity and cancer risk across various cancer types.

**Table 1:** Meta-analysis data on the association between obesity and cancer risk

Author, Year	Meta-analysis title	Outcomes	Conclusions
Reneham, 2008 [74]	Body-mass index and incidence of cancer: a systematic review and meta-analysis of prospective observational studies	1. In men, a 5 kg/m^2^ increase in BMI was strongly associated with esophageal adenocarcinoma (RR 1.52, p < 0.0001) and with thyroid (1.33, p = 0.02), colon (1.24, p < 0.0001), and renal (1.24, p < 0.0001) cancers 2. In women, strong associations between a 5 kg/m^2^ increase in BMI and endometrial (1.59, p < 0.0001), gallbladder (1.59, p = 0.04), esophageal adenocarcinoma (1.51, p < 0.0001), and renal (1.34, p < 0.0001) cancers	1. ↑ BMI is associated with ↑ risk of common and less common cancers 2. For some cancer types, associations differ between genders and populations of different ethnic origins
Cao, 2011 [111]	Body Mass Index, Prostate Cancer–Specific Mortality, and Biochemical Recurrence: a Systematic Review and Meta-analysis	1. Among the six population-based cohort studies in 1,263,483 initially cancer-free men, 6,817 prostate cancer deaths occurred; a 5 kg/m^2^ increase in BMI was associated with 15% (RR: 1.15, 95% CI: 1.06–1.25, P < 0.01) higher risk of dying of prostate cancer 2. In the six post-diagnosis survival studies on 18,203 patients with 932 prostate cancer deaths, a 5 kg/m^2^ increase in BMI was associated with 20% higher prostate cancer-specific mortality (RR: 1.20, 95% CI: 0.99–1.46, P = 0.06) 3. In the sixteen studies which followed 26,479 prostate cancer patients after primary treatment, a 5 kg/m^2^ increase in BMI was significantly associated with 21% increased risk of biochemical recurrence (RR: 1.21, 95% CI: 1.11–1.31 P < 0.01)	↑ BMI is associated with risk of prostate cancer-specific mortality in prospective cohort studies and biochemical recurrence in prostate cancer patients
Chen, 2012 [75]	Excess body weight and the risk of primary liver cancer: An updated meta-analysis of prospective studies	BMI ≥ 25 kg/m^2^ and obesity (BMI ≥ 30 kg/m^2^) were associated with an ↑ risk of primary liver cancer, with significant heterogeneity (EBW: SRRs 1.48, 95% CIs 1.31–1.67, P(h) < 0.001; Obesity: SRRs 1.83, 95% CIs 1.59–2.11, P(h) < 0.001	Excess body weight or obesity is associated with an ↑ risk of primary liver cancer in both men and women
Chen, 2013 [76]	Body Mass Index and Risk of Gastric Cancer: A Meta-analysis of a Population with More Than Ten Million from 24 Prospective Studies	1. BMI ≥ 30 kg/m^2^ was associated with an ↑ risk of gastric cancer (OR = 1.13, 95% CI = 1.03–1.24) compared with normal weight (BMI = 18.5 to < 25 kg/m^2^), while overweight (BMI < 30 kg/m^2^) showed no association (OR = 1.04, 95% CI = 0.96–1.12) 2. Stratified analysis showed there were associations between obesity and the ↑ risk of gastric cancer for males (OR = 1.27, 95% confidence interval = 1.09–1.48), non-Asians (OR = 1.14, 95% CI = 1.02–1.28) and both cohort studies (OR = 1.10, 95% CI = 1.00–1.22) and case–control studies (OR = 1.29, 95% CI = 1.03–1.60). Both overweight (OR = 1.22, 95% CI = 1.05–1.42) and obesity (OR = 1.61, 95% CI = 1.15–2.24) were associated with the ↑ risk of gastric cardia cancer	↑ BMI was positively associated with the risk of gastric cardia cancer but not with gastric non-cardia cancer
Chan, 2014 [99]	Body mass index and survival in women with breast cancer-systematic literature review and meta-analysis of 82 follow-up studies	1. For BMI before diagnosis, compared with normal weight women, RRs of total mortality were 1.41 [95% CI 1.29–1.53] for obese (BMI > 30.0), 1.07 (95% CI 1.02–1.12) for overweight (BMI = 25.0- < 30.0) and 1.10 (95% CI 0.92–1.31) for underweight (BMI < 18.5) women 2. For obese women, the summary RRs were 1.75 (95% CI 1.26–2.41) for pre-menopausal and 1.34 (95% CI 1.18–1.53) for post-menopausal breast cancer. For each 5 kg/m^2^ increment of BMI before, < 12 months after, and ≥ 12 months after diagnosis, increased risks of 17%, 11%, and 8% for total mortality, and 18%, 14%, and 29% for breast cancer mortality were observed, respectively	Obesity is associated with poorer overall, and breast cancer survival in pre- and post-menopausal breast cancer, regardless of when BMI is ascertained
Playdon, 2015 [101]	Weight Gain After Breast Cancer Diagnosis and All-Cause Mortality: Systematic Review and Meta-Analysis	1. Weight gain ≥ 5.0% compared with maintenance < ± 5.0% was associated with ↑ all-cause mortality (HR = 1.12, 95% CI = 1.03 to 1.22, P = 0.01, I(2) = 55%) 2. Higher risk of mortality was apparent for weight gain ≥ 10% (HR = 1.23, 95% CI = 1.09 to 1.39, P < 0.001); 5% to 10.0% weight gain was not associated with all-cause mortality (P = 0.40) 3. The association was not statistically significant for those with a pre-diagnosis BMI of less than 25 kg/m^2^ (HR = 1.14, 95% CI = 0.99 to 1.31, P = 0.07) or with a BMI of 25 kg/m^2^ or higher (HR = 1.00, 95% CI = 0.86 to 1.16, P = 0.19) 4. Weight gain of 10% or more was not associated with hazard of breast cancer-specific mortality (HR = 1.17, 95% CI = 1.00 to 1.38, P = 0.05)	1. Weight gain after diagnosis of breast cancer is associated with ↑ all-cause mortality rates compared with maintaining body weight 2. Adverse effects are ↑ for weight gains of 10% or higher
Jenabi, 2015 [79]	The effect of body mass index on endometrial cancer: a meta-analysis	The estimated RR and OR of endometrial cancer was 1.34 (95% CI: 1.20, 1.48) and 1.43 (95% CI: 1.30, 1.56) for the overweight and 2.54 (95% CI: 2.27, 2.81) and 3.33 (95% CI: 2.87, 3.79) for the obese, respectively	BMI is strongly associated with an ↑ risk of endometrial cancer
Lee, 2015 [105]	Association between Body Mass Index and Prognosis of Colorectal Cancer: A Meta Analysis of Prospective Cohort Studies	1. Underweight before cancer diagnosis was associated with ↑ all-cause mortality (RR: 1.63, 95% CI: 1.18–2.23, p < 0.01) and being obese (BMI ≥ 30 kg/m2) before cancer diagnosis was associated with ↑ CRC-specific mortality (RR: 1.22, 95% CI: 1.003–1.35, p < 0.01) and all-cause mortality (RR: 1.25, 95% CI: 1.14–1.36, p < 0.01) 2. Being underweight (RR: 1.33, 95% CI: 1.20–1.47, p < 0.01), obese (RR: 1.08, 95% CI: 1.03–1.3, p < 0.01), and class II/III obese (BMI ≥ 35 kg/m2; RR: 1.13, 95% CI: 1.04–1.23, p < 0.01) after diagnosis were associated with significantly ↑ all-cause mortality	Obesity prior to diagnosis of CRC was associated with ↑ CRC-specific mortality and all-cause mortality, whereas being obese after diagnosis was associated with ↑ all-cause mortality
Majumder, 2015 [107]	Premorbid Obesity and Mortality in Patients With Pancreatic Cancer: A Systematic Review and Meta-analysis	1. Each 1 kg/m^2^ ↑ in BMI was associated with 10% ↑ in mortality (aHR, 1.10; 95% CI, 1.05–1.15) with minimal heterogeneity 2. In the subgroup analysis, obesity was associated with ↑ mortality in Western populations (11 studies; aHR, 1.32; 95% CI, 1.22–1.42) but not in Asia–Pacific populations (2 studies; aHR, 0.98; 95% CI, 0.76–1.27)	Association between ↑ level of obesity with ↑ mortality in patients with pancreatic cancer in Western but not Asia–Pacific populations
Aune, 2016 [88]	BMI and all-cause mortality: systematic review and non-linear dose–response meta-analysis of 230 cohort studies with 3.74 million deaths among 30.3 million participants	The SRR for a 5-unit increment in BMI was 1.18 (95% CI 1.15 to 1.21; n = 44) among never smokers, 1.21 (1.18 to 1.25; n = 25) among healthy never smokers, 1.27 (1.21 to 1.33; n = 11) among healthy never smokers with exclusion of early follow-up, and 1.05 (1.04 to 1.07; n = 198) among all participants	Overweight and obesity is associated with ↑ risk of all-cause mortality and the nadir of the curve was observed at BMI 23–24 kg/m^2^ among never smokers, 22–23 kg/m^2^ among healthy never smokers, and 20–22 kg/m^2^ with longer durations of follow-up
Secord, 2016 [110]	Body mass index and mortality in endometrial cancer: A systematic review and meta-analysis	1. According to a random-effects meta-analysis, patients with endometrial cancer had noticeably greater odds of dying as their BMI increased 2. In particular, the odds ratios for the BMI categories of 25–29.9, 30–34.9, 35–39.9, and 40 + were 1.01, 1.17, 1.26, and 1.66, respectively 3. When comparing endometrial cancer patients with a BMI ≥ 40 to those with a BMI < 25, the odds ratio for all-cause mortality was 1.66 (CI: 1.10–2.51, p = 0.02) 4. According to a single dose–response model, the odds of all-cause mortality increased by 9–2% for every 10% increase in BMI (p = 0.007)	In women with endometrial cancer, a ↑ BMI is substantially linked to a ↑ all-cause mortality rate; those with a BMI ≥ 40 are at the highest risk
Doleman, 2016 [104]	Body mass index and colorectal cancer prognosis: a systematic review and meta-analysis	1. Obese patients had an ↑ risk of all-cause mortality (RR 1.14; 95% CI 1.07–1.21), cancer-specific mortality (RR 1.14; 95% CI 1.05–1.24), recurrence (RR 1.07; 95% CI 1.02–1.13) and worse disease-free survival (RR 1.07; 95% CI 1.01–1.13) 2. Underweight patients also had an ↑ risk of all-cause mortality (RR 1.43; 95% CI 1.26–1.62), cancer-specific mortality (RR 1.50; 95% CI 1.20–1.87), recurrence (RR 1.13; 95% CI 1.05–1.21) and worse disease-free survival (RR 1.27; 95% CI 1.13–1.43)	Both obese and underweight patients with CRC have an ↑ risk of all-cause mortality, cancer-specific mortality, disease recurrence and worse disease-free survival compared to normal weight patients
Mintziras, 2018 [106]	Sarcopenia and sarcopenic obesity are significantly associated with poorer overall survival in patients with pancreatic cancer: Systematic review and meta-analysis	1. A significantly lower overall survival rate was linked to sarcopenia (HR 1.49; 95% CI 1.27–1.74, p < 0.001) 2. A significantly lower overall survival rate was linked to sarcopenic obesity, which was reported in 0.6% to 25.0% of patients (HR 2.01; 95% CI 1.55–2.61, p < 0.001) 3. The incidence of major complications ranged from 8.6% to 33.9%	Sarcopenia and sarcopenic obesity are significantly associated with poorer overall survival in patients with pancreatic cancer
Petrelli, 2021 [54]	Association of Obesity With Survival Outcomes in Patients With Cancer A Systematic Review and Meta-analysis	1. Obesity was associated with a ↓ OS (HR, 1.14; 95% CI, 1.09–1.19; P < 0.001) and CSS (HR, 1.17; 95% CI, 1.12–1.23; P < 0.001) 2. Patients were also at ↑ risk of recurrence (HR, 1.13; 95% CI, 1.07–1.19; P < 0.001) 3. Patients with obesity and lung cancer, renal cell carcinoma, or melanoma had better survival outcomes compared with patients without obesity and the same cancer (lung: HR, 0.86; 95% CI, 0.76–0.98; P = 0.02; renal cell: HR, 0.74; 95% CI, 0.53–0.89; P = 0.02; melanoma: HR, 0.74; 95% CI, 0.57–0.96; P < 0.001)	Obesity was associated with ↑ mortality overall in patients with cancer. However, patients with obesity and lung cancer, renal cell carcinoma, and melanoma had a ↓ risk of death than patients with the same cancers without obesity
Lazzeroni, 2021 [84]	A Meta-Analysis of Obesity and Risk of Colorectal Cancer in Patients with Lynch Syndrome: The Impact of Sex and Genetics	1. A twofold risk of CRC was found in obese men compared to non-obese men (SRR = 2.09; 95%CI: 1.23–3.55), and no indication of publication bias (p = 0.13) 2. For women, there was no discernible ↑ risk associated with obesity 3. Subjects with an *MLH1* mutation had a 49% higher risk of CRC due to obesity (SRR = 1.49; 95% CI: 1.11–1.99)	Public initiatives to lower overweight in individuals with Lynch syndrome, especially in men, are supported by the distinct effects of sex on obesity and CVD risk
Rivera-Izquierdo, 2021 [112]	Obesity as a Risk Factor for Prostate Cancer Mortality: A Systematic Review and Dose–Response Meta-Analysis of 280,199 Patients	1. Obesity was associated with ↑ PC-specific mortality (HR: 1.19, 95% CI: 1.10–1.28) and all-cause mortality (HR: 1.09, 95% CI: 1.00–1.18) 2. There was a 9% ↑ (95% CI: 5–12%) in PC-specific mortality and 3% ↑ (95% CI: 1–5%,) in all-cause mortality per 5 kg/m^2^ increase in BMI	Obesity was associated with ↑ prostate cancer -specific mortality
Mie Thu Ko, 2024 [95]	The Association Between Obesity and Malignant Progression of Barrett’s Esophagus: A Systematic Review and Dose- Response Meta-Analysis	Each 5-kg/m^2^ increase in BMI was associated with a 6% increase in the risk of malignant progression (adjusted OR, 1.06; 95% CI, 1.02–1.10; p < 0.001). There was no significant difference in pooled annual rate of progression between males and females (p = 0.15)	Obesity as measured by BMI is associated with ↑ malignant progression of Barrett’s esophagus with a dose–response relationship

Abbreviations: BMI: Body Mass Index; CRC: Colorectal Cancer. EBW: Excess Body Weight, PC: Prostate Cancer, CI: Confidence Interval, RR: Risk Ratio, HR: Hazard Ratio, OR: Odds Ratio SRR = Summary Relative Risk

### Ultra-Processed Food Consumption and Its Association with Cancer Risk: Where Do We Stand?

#### Epidemiological Evidence

The bulk of evidence supporting the link between UPF consumption and cancer risk originates from epidemiological studies assessing the relationship between consumption of particular food groups and cancer incidence, as the food processing frameworks have only recently been established [114]. In 2018, the World Cancer Research Fund conducted a comprehensive review of recent international studies on diet and cancer risk, including an assessment of food preservation and processing methods. The Continuous Update Project (CUP) serves as the primary global repository for scientific research on nutrition, physical activity, and diet-based cancer prevention and survivorship [115].

In a prospective cohort analysis, researchers recruited 40-years-old to 69-years-old UK Biobank participants (N = 197.426, 54.6% women) who completed 24-h dietary recalls between 2009 and 2012 and were monitored until January 31, 2021 [29]. Using the NOVA food classification system, food items were categorized based on how much they had been processed. The percentage of total food intake (g/day) that each individual consumed as UPFs was quantified. They used proportional hazards models to assess potential associations after controlling for baseline sociodemographic factors, alcohol consumption, smoking status, physical activity, BMI, and total energy intake. They found that the average consumption of UPFs across the entire diet was 22.9% ± 13.3%. Four thousand hundred and nine cancer-related deaths and 15,921 cancer cases were reported over a median follow-up period of 9.8 years. An increased incidence of ovarian cancer HR 1.19; 95% CI, 1.08 to 1.30) and overall cancer (HR, 1.02; 95% CI, 1.01 to 1.04) was linked to every 10% increase in UPFs consumption. Additionally, a 10% increase in UPFs consumption was linked to a higher risk of death from cancer-related causes, such as breast (HR, 1.16; 95% CI, 1.02 to 1.32), ovarian (HR, 1.30; 95% CI, 1.13 to 1.50), and overall (HR, 1.06; 95% CI, 1.03 to 1.09) [5].

In another systematic review, researchers integrated data from nine case control studies and four prospective cohort studies to fully quantify the relationship between UPFs consumption and different cancer risk types [116]. Their results revealed a correlation between higher UPF intake and a higher risk of breast and colorectal cancer. For every 10% increase in UPFs in the diet, the risk of colorectal cancer rose by 4%. Subgroup analysis further indicated a substantial association between UPF consumption and elevated colorectal cancer risk in men, while this correlation was absent in women. By showing that a high intake of UPFs was linked to an increased risk of some site-specific cancers, particularly those of the digestive tract, as well as some hormone-related cancers, such as colorectal and breast cancers, this systematic review added depth to our understanding of the possible implications of a processed diet on the development of cancer [29].

In a systematic review, scientists associated trans-fatty acid intake with any cancer risk [28]. The meta-analyses on total trans-fat showed a significant positive association for prostate cancer (odds ratio [OR] 1.49; 95%CI, 1.13–1.95) and colorectal cancer (OR 1.26; 95%CI, 1.08–1.46) but not for breast cancer (OR 1.12; 95%CI, 0.99–1.26), ovarian cancer (OR 1.10; 95%CI, 0.94–1.28), or non-Hodgkin lymphoma (OR 1.32; 95%CI, 0.99–1.76). Findings varied by fatty acid subtype, with some partially hydrogenated vegetable oils even showing cancer-preventive properties. The positive trans-fat-cancer relationship was enhanced by the following moderators: European ancestry, menopause, older age, overweight, and gender (the direction was cancer-site specific) [28].

In another systematic literature search for observational studies, researchers investigated the association between cancer risk and UPF consumption in 11 reports [21]. Three prospective cohorts and eight retrospective case–control studies were among the eleven reports that were found. The result was the possibility of developing colorectal, breast, prostate, pancreatic, chronic lymphocytic leukemia, and central nervous system tumors, as well as the possibility of developing cancer in its entirety. After controlling for confounding variables like obesity and total energy intake, nine studies found a significant positive correlation between UPF intake and all evaluated cancers except prostate cancer. An increased risk of breast cancer (HR = 1.11, 95% CI 1.01 to 1.21) and overall cancer (HR = 1.13, 95% CI 1.07 to 1.18) was linked to a 10% increase in the diet's UPF content. Tumors of the central nervous system and chronic lymphocytic leukemia showed less strong correlations. Some of the studies' common limitations included higher case participation rates, the possibility that some foods were incorrectly classified as UPF or non-UPF, and the lack of a diet assessment prior to a known diagnosis (the case–control studies) [21].

In a systematic review and dose–response meta-analysis study, researchers investigate the possibility of a link between high UPF consumption and breast cancer risk [26]. In total, six publications with 462,292 participants qualified for this analysis. A higher risk of breast cancer was associated with the highest UPF consumption as compared to the lowest (RR = 1.10; 95% CI: 1.00–1.22, p = 0.056). Additionally, the linear dose–response analysis revealed that a 5% increased risk of breast cancer was associated with every 10% increase in UPF consumption (RR = 1.05; 95% CI: 1.00–1.10, p = 0.048). According to subgroup analyses, UPF use was positively correlated with the risk of breast cancer in case–control studies (RR = 1.13; 95% CI: 1.01–1.26, p = 0.028). A significant positive correlation was also found between UPF consumption and breast cancer risk in the subgroup with a sample size of less than 5,000 (RR = 1.17; 95% CI: 1.02–1.35, p = 0.028). Their findings suggest that there is a slight correlation between increased UPF consumption and an increased risk of breast cancer [26].

In a separate study, researchers assessed the meta-analytic evidence that linked exposure to UPFs as defined by the Nova food classification system to negative health consequences [23]. 45 distinct pooled analyses were found by the search, comprising 32 non-dose–response associations and 13 dose–response associations (n = 9,888,373). Exposure to UPFs was found to be directly associated with 32 (71%) health parameters, including mortality, cancer, and outcomes related to mental, respiratory, cardiovascular, gastrointestinal, and metabolic health [23].

The purpose of another systematic review and meta-analysis of prospective cohort studies was to look into the relationship between the risk of gastrointestinal cancer and UPF use [22]. When compared to the lowest UPF intake, researchers discovered that the highest UPF consumption was significantly linked to an increased risk of colorectal cancer (HR 1.11; 95% CI 1.03–1.21; P = 0.01), colon cancer (HR 1.12; 95% CI 1.02–1.23; P = 0.02), and non-cardia gastric cancer (HR 1.43; 95% CI 1.02–2.00; P = 0.04). Hepatocellular, esophageal, pancreatic, gastric cardia, and rectal cancers were not linked to high UPF consumption, though [22].

Barbaresko et al. provide an overview of the existing evidence of UPFs on human health. They conducted a systematic search in four databases until January 2024. Moderate certainty of evidence was found for all-cause mortality (RR per 50 g: 1.02; 95% CI: 1.01, 1.03), cardiovascular disease incidence and mortality (per 50 g/d: 1.04; 95% CI: 1.02, 1.06, and 1.05; 95% CI: 1.01, 1.08), T2DM incidence (per 10%: 1.12; 95% CI: 1.10, 1.13) and colorectal cancer (per 10%: 1.04; 95% CI: 1.01, 1.07). Similar estimates for a number of outcomes, including inflammatory bowel disease, obesity, metabolic syndrome, nonalcoholic fatty liver disease, mental health, and nutritional quality, were noted; however, the level of evidence certainty was low. When it comes to the NOVA concept, it's still unclear if eating UPFs is a sign of a poor diet or if food processing raises health risks [24].

In conclusion, there is a steady and substantial association between UPF use and the risk of a number of cancers, including colorectal, prostate, breast, non-cardia gastric, and pancreatic cancers, according to the present data. The public, decision-makers, and revised dietary guidelines may benefit from these findings in terms of improving public health. Large prospective cohort studies in particular need to be conducted in order to validate these findings. Additionally, in order to improve human health, these findings support the development and assessment of population-based and public health interventions that aim to target and lower dietary exposure to UPFs. Additionally, they advise and assist with urgent mechanistic research. Table 2 presents an overview of recent meta-analyses examining the association between the consumption of UPFs and the risk of malignancy.

**Table 2:** Meta-Analysis summary of the association between ultra-processed food consumption and cancer risk

Author, Year	Meta-analysis title	Outcomes	Conclusions
Lian, 2023 [29]	Association between ultra-processed foods and risk of cancer: a systematic review and meta-analysis	The highest UPFs consumption was linked with increased risk of CRC (OR = 1.23, 95% CI: 1.10–1.38), colon cancer (OR = 1.25, 95% CI: 1.14–1.36), and breast cancer (OR = 1.10, 95% CI: 1.00–1.20) but not rectal cancer (OR = 1.18, 95% CI: 0.97–1.43) and prostate cancer (OR = 1.03, 95% CI: 0.93–1.12)	↑ UPF consumption is associated with a significant risk of certain site-specific cancers, especially the digestive tract and some hormone-related cancers
Isaksen, 2023 [21]	Ultra-processed food consumption and cancer risk: A systematic review and meta-analysis	Raising the amount of UPF in the diet by 10% was linked to a higher risk of breast cancer (HR = 1.11, 95% CI 1.01 to 1.21) and overall cancer (HR = 1.13, 95% CI 1.07 to 1.18)	↑ consumption of UPF has been consistently linked to a significant risk of developing various cancers, including CRC, breast, and pancreatic cancers
Shu, 2023 [26]	Association between ultra-processed food consumption and risk of breast cancer: a systematic review and dose–response meta-analysis of observational studies	1. A higher risk of breast cancer was associated with the highest UPF consumption compared to the lowest (RR = 1.10; 95% CI: 1.00–1.22, p = 0.056) 2. The linear dose–response analysis revealed that a 5% increased risk of breast cancer was associated with every 10% increase in UPF consumption (RR = 1.05; 95% CI: 1.00–1.10, p = 0.048)	↑ consumption of UPF is slightly related to a higher risk of breast cancer
Lane, 2024 [23]	Ultra-processed food exposure and adverse health outcomes: umbrella review of epidemiological meta-analyses	Highly suggestive evidence indicated that greater exposure to UPFs was directly associated with HR of incident all-cause mortality (RR 1.21, 1.15 to 1.27; low)	↑ exposure to UPFs was associated with a higher risk of adverse health outcomes, especially cardiometabolic, common mental disorder, cancer and mortality outcomes
Meine, 2024 [22]	Ultra-Processed Food Consumption and Gastrointestinal Cancer Risk: A Systematic Review and Meta-Analysis	The highest UPF consumption was significantly associated with an increased risk of CRC (HR 1.11; 95% CI 1.03–1.21; P = 0.01), colon cancer (HR 1.12; 95% CI 1.02–1.23; P = 0.02), and non-cardia gastric cancer (HR 1.43; 95% CI 1.02–2.00; P = 0.04) compared with the lowest UPF intake	↑ UPF consumption was substantially linked to both non-cardia gastric cancer and CRC
Barbaresko, 2024 [24]	Ultra-processed food consumption and human health: an umbrella review of systematic reviews with meta-analyses	Moderate certainty of evidence was found for all-cause mortality (RR per 50 g: 1.02; 95% CI: 1.01, 1.03), CVD incidence and mortality (per 50 g/d: 1.04; 95% CI: 1.02, 1.06, and 1.05; 95% CI: 1.01, 1.08), T2DM incidence (per 10%: 1.12; 95% CI: 1.10, 1.13) and CRC (per 10%: 1.04; 95% CI: 1.01, 1.07)	It's still unclear, when it comes to the NOVA concept, whether eating UPFs is a sign of a poor diet or if food processing raises health risks

Abbreviations: UPFs: Ultra-Processed Foods, CI: Confidence Interval, T2DM: Type 2 Diabetes Mellitus, RR: Risk Ratio, HR: Hazard Ratio, OR: Odds Ratio.

### Limitations and Challenges of Epidemiological Studies Examining the Association Between UPFs and Cancer Risk

Future cohort studies and diverse settings are needed to confirm the positive associations found in previously published studies assessing the relationship between UPF intake and cancer risk [27, 49]. There were some contradictory results, which may be due to the lack of standardization in the definition and classification of UPFs. To facilitate comparison with other studies and consideration of the entire food system, future research should aim to use a valid and standardized framework for classifying food processing.

Studies examining the link between reduced food processing and cancer risk are also needed. Understanding the relationship between changes in the level of food processing in diet and cancer risk can also be improved through longitudinal data with repeated measures of dietary intake. There is also a lack of studies examining the possible mediating effects of each component – overconsumption and weight gain, diet quality, contaminants in food processing, food additives, etc. in the associations between consumption of UPFs and cancer risk. Substitution analyzes and short-term nutritional interventions to evaluate substitution for specific groups (e.g., minimally processed meat) will also be important to establish public health guidelines.

Because epidemiological studies have so far produced conflicting results, further research is needed to fully understand the role of chemical compounds in the association between UPFs and cancer risk. This applies, for example, to artificial sweeteners as well as nitrates and nitrites. In addition, further research is needed to understand how the food matrix and additives undergo chemical structural changes due to physical, chemical and enzymatic reactions during processing. It is also important to examine the synergistic effects of multiple additives in the same product. Both in vitro and in vivo experiments as well as the collection of comprehensive data on the consumption of highly processed foods can be used to investigate this synergistic effect. Additionally, searching for biological markers associated with each additive may be a promising approach as it circumvents the recall bias and social desirability bias associated with reporting the amount of UPF consumed [27, 49]. Targeted experimental studies are urgently needed to examine the cumulative effect of all the proposed putative mechanisms involved in these associations between UPF consumption and cancer risk.

### Potential Biological Mechanisms Linking UPF Consumption to Cancer Risk

The consumption of UPFs may potentially raise cancer risk due to their obesogenic qualities and the presence of various contaminants and food additives. Compared to minimally processed foods, UPFs typically offer lower nutritional quality, as demonstrated in multiple studies [5, 116, 117]. Diets high in UPFs typically contain lower levels of dietary fiber, protein, sodium, and potassium, while exhibiting higher energy density and increased levels of free sugars, total fat, saturated fat, and trans-fat [5, 117]. A study by Hall et al. further indicates that individuals consuming UPF-rich diets tend to consume more calories and gain more weight than those on unprocessed diets [43]. Excess calorie consumption, predominantly from fats and carbohydrates, underscores the hyper-palatable and appealing nature of UPF products, which has been linked to obesity, a known risk factor for at least 13 cancer types [56, 118].

Food contaminants, particularly those formed during processing, are also thought to contribute to the cancer risk associated with UPF consumption. When foods are processed, they undergo various physical, chemical, and biological transformations, leading to changes in their chemical structure and the formation of "neo-formed" processing contaminants. These include substances like trans-fats and acrylamide, the latter forming when the amino acid asparagine reacts with glucose or fructose under high heat. Elevated consumption of industrial trans-fats, for instance, has been associated with increased cancer risk [28, 119, 120]. Additional contaminants linked to increased cancer risk include oxyhalides, haloacetic acids, heterocyclic amines, and polycyclic aromatic hydrocarbons [121].

UPFs may also contain “indirect” contaminants derived from packaging materials, which can disrupt hormonal pathways [122]. For instance, UPFs have been positively correlated with urinary concentrations of Di(ethylhexyl) phthalate (DEHP), an endocrine disruptor chemical that is widely used as a plasticizer [123]. Research on both animals and humans has demonstrated that DEHP exposure may promote carcinogenesis through various molecular mechanisms, including DNA damage [124]. Recent findings suggest that DEHP exposure could increase cancer stemness, facilitating colon cancer metastasis [123]. Similarly, bisphenol-A (BPA), another endocrine disruptor found in many food packaging types has been associated with elevated cancer risk [49, 126, 127]. BPA exposure disrupts multiple signaling pathways, contributing to cancer development and the etiopathogenesis of obesity, as evidenced by epidemiological, animal, and mechanistic studies, including meta-analyses [49, 57, 128]. Additionally, obesity-related conditions such as insulin resistance, T2DM, dyslipidemia, hypertension, and hormonal imbalances have been linked to common chemicals like BPA, phthalates, and their analogs [49, 129, 130].

**Fig. 2:**
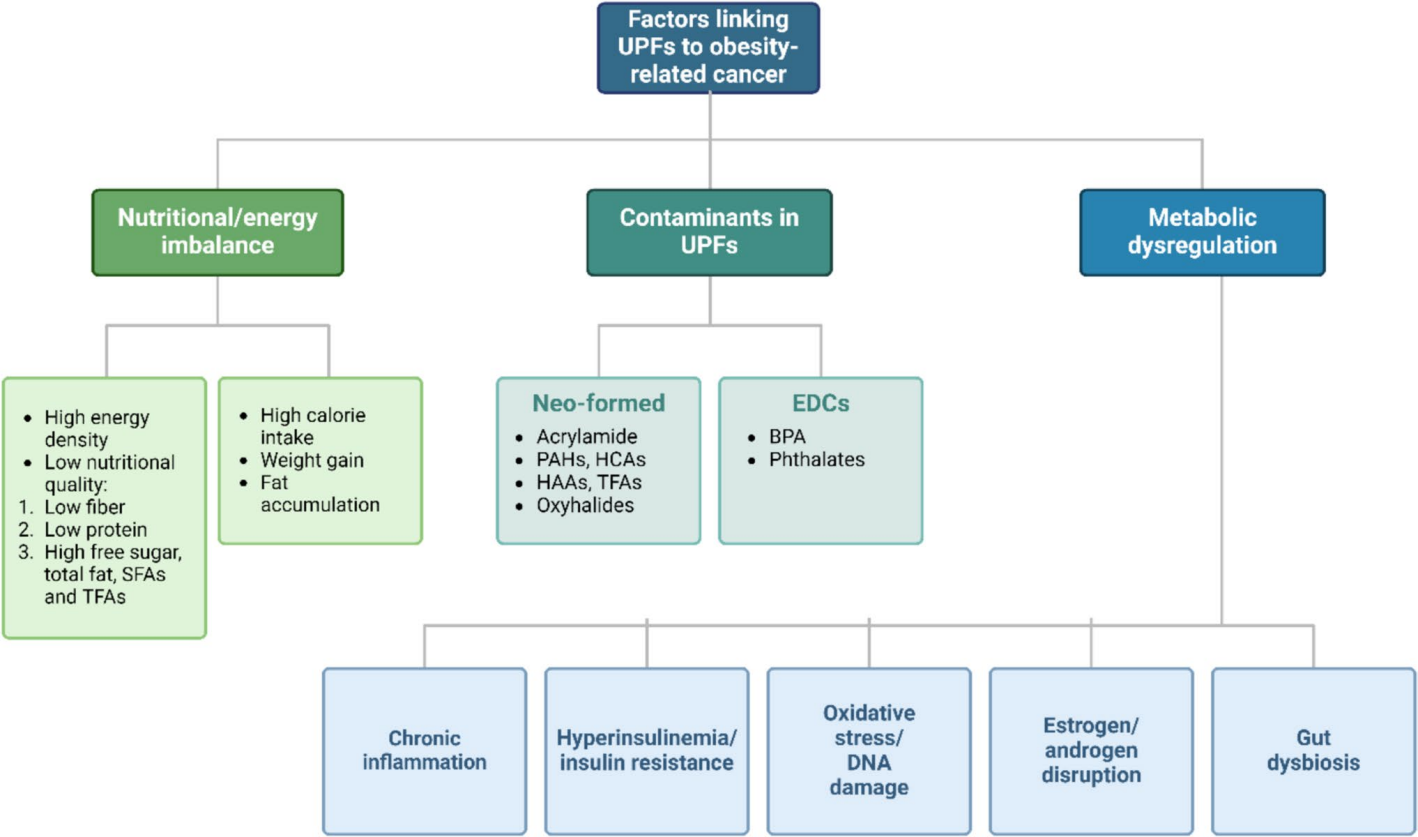
Key factors and mechanisms linking ultra-processed foods to obesity-related cancer. Abbreviations: BPA: Bisphenol A; HAAs: Heterocyclic Aromatic Amines; HCAs: Heterocyclic Amines; PAHs: Polycyclic Aromatic Hydrocarbons; SFAs: Saturated Fatty Acids; TFAs: Trans Fatty Acids; UPFs: Ultra-Processed Foods. Created in BioRender. Kounatidis, D. (2024) https://BioRender.com/w42t709. Assessed on 14 December 2024

Beyond recognized endocrine-disrupting chemicals, numerous chemical compounds or complex mixtures have been identified as agents that disrupt hormone and growth factor signaling, metabolism, and carcinogenic processes. These compounds, often found in plastic bottles, can dysregulate the IGF-1 axis and exhibit anti-estrogenic or anti-androgenic effects, further contributing to cancer risk [49, 131]. Recent research has highlighted the potential role of intestinal microbiota in obesity-related cancer risk and the association between UPF consumption and microbiota alterations. Thus, the correlation between UPF intake and obesity raises the hypothesis that gut dysbiosis may contribute to obesity-related carcinogenesis prompted by high levels of UPF consumption [132, 133]. Figure 2 presents the main factors linking UPFs to obesity-related cancer.

### The Intricate Relationship Between Ultra-Processed Foods and the Gut Microbiome: Implications for Early-Onset Colorectal and Gastric Cancer

The gut microbiome encompasses the collective genome of microorganisms residing in the gut, including bacteria, viruses, fungi, and archaea. These microorganisms, often termed the gut microbiota, play essential roles in host health, as confirmed by metabolomics studies. Under normal conditions, a homeostatic balance exists between the gut microbiota and the host. Disruption of this balance, however, leads to a state known as gut dysbiosis [134]. Gut dysbiosis arises from alterations in the intestinal epithelial barrier, resulting in compromised barrier integrity, commonly referred to as "leaky gut." This leaky gut permits pathogenic microbes and toxins from the gut lumen to enter the systemic circulation, creating a state of endotoxemia, marked by increased lipopolysaccharide (LPS) levels from gram-negative bacteria in the bloodstream. Consequently, this breach fosters an inflammatory environment through the upregulation of pro-inflammatory cytokines, perpetuating inflammation and driving shifts in the diversity and composition of the gut microbiota [135].

UPFs contain additives, such as emulsifiers like carboxymethylcellulose (CMC) and polysorbate-80 (P-80), known to disrupt the intestinal epithelial barrier. CMC and P-80 have been shown to impair tight junction (TJ) proteins and reduce mucus production, contributing to a "leaky gut" [132, 136]. Naimi et al. evaluated the effects of 20 emulsifiers on gut microbiota composition and observed that polysorbate-80, carrageenan, and agar–agar decreased levels of *Faecalibacterium* and *Akkermansia*, both of which are recognized for their anti-inflammatory properties. Additionally, carrageenan, glyceryl stearate, sorbitan monostearate, and xanthan gum increased LPS levels, leading to metabolic endotoxemia. Their findings suggest that most-but not all-emulsifiers cause detrimental shifts in gut microbiota composition, promoting inflammation and endotoxemia [137].

Miclotte et al. investigated five emulsifiers: two synthetic (CMC and P-80), two biotechnological (sophorolipids and rhamnolipids), and one natural (soy lecithin). They found that CMC, P-80, sophorolipids, and rhamnolipids reduced gut microbiota diversity, while soy lecithin did not show this effect [138]. Carrageenan, widely considered a safe emulsifier derived from red seaweeds, has also been shown to disrupt zona occludens-1 (ZO-1), a critical TJ component [139]. Interestingly, P-80 enhances the absorption of DEHP, a plasticizer used in food packaging, and its toxic metabolite mono-2-ethylhexyl phthalate (MEHP), both of which detrimentally alter gut microbiota composition, reduce microbial diversity, and disrupt TJ integrity, further exacerbating metabolic endotoxemia and inflammation [140]. Therefore, emerging evidence links UPFs to alterations in gut microbiota and intestinal barrier disruption, resulting in chronic low-grade inflammation and endotoxemia, both associated with weight gain, obesity, metabolic syndrome, and potentially obesity-related cancers [141, 142].

Early-onset colorectal cancer (EOCRC)-defined as colorectal cancer occurring before age 50-illustrates the potential impact of UPFs. Titanium dioxide (E171), a whitening additive used extensively in UPFs, has been FDA-approved since 1966 under the assumption that it remains under 1% of food weight. However, its use has markedly increased, with children under 10 experiencing greater exposure levels than adults in the U.S. and UK [143]. Given that obesity and gut dysbiosis are known EOCRC risk factors, E171 might play a contributory role [144]. Additionally, *Fusobacterium nucleatum*, linked to colorectal cancer, exemplifies how gut dysbiosis may promote colon carcinogenesis [145]. Besides inflammation, other pathways, such as β-catenin signaling, may also play roles in this process [146]. Gastric cancer, another malignancy that is not associated with obesity, but is strongly linked to *Helicobacter pylori (H.pylori)* infection [147]. Only recently, Ebrahimi et al. reported a correlation between increased UPF consumption and heightened *H. pylori* infection risk. Their study, involving 150 infected patients and 302 controls, called for additional large-scale research to substantiate these findings [148].

**Fig. 3:**
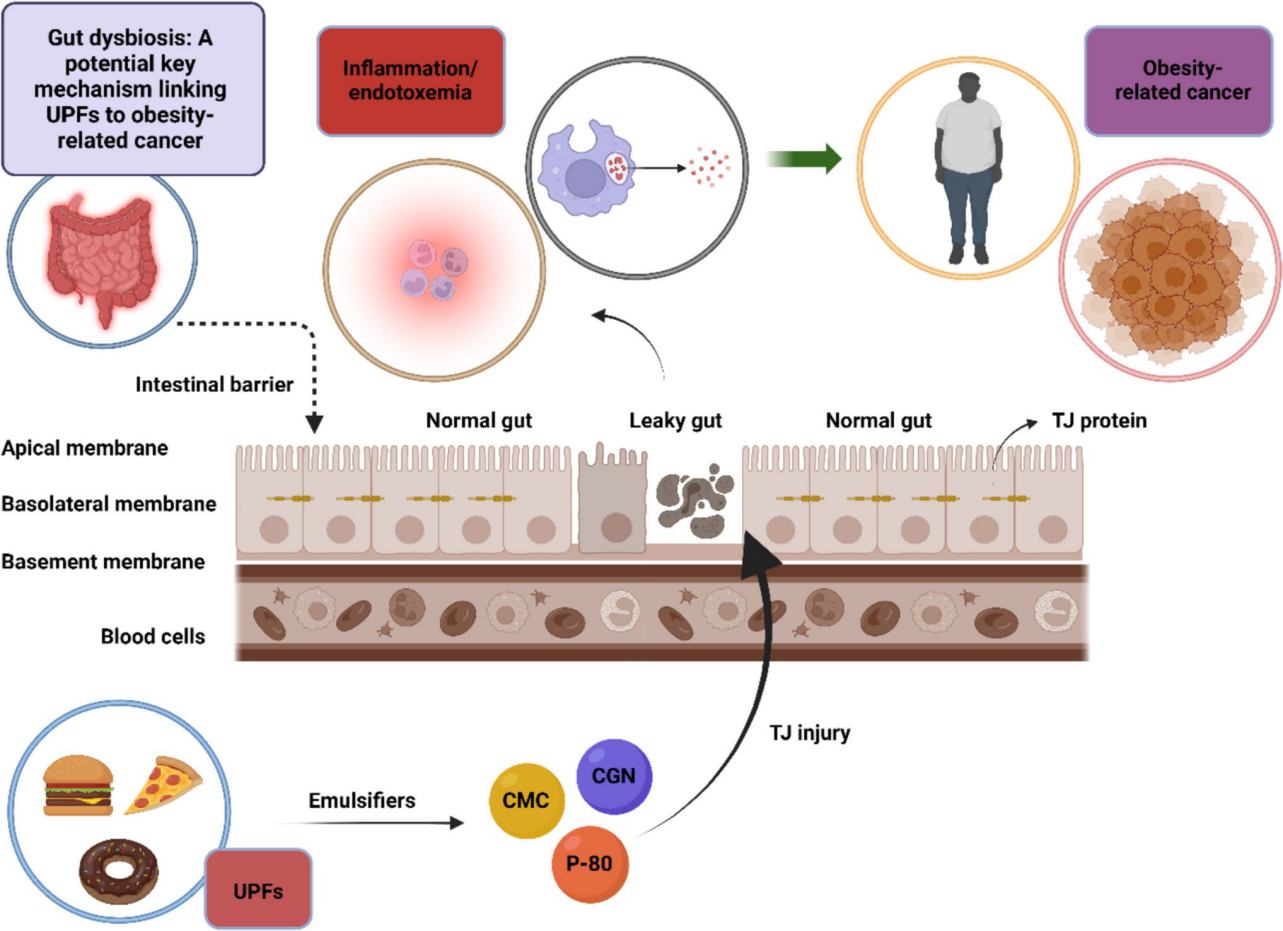
From UPF overconsumption to obesity-associated cancer: The potential role of gut dysbiosis. UPFs contain emulsifiers such as CMC, P-80, and CGN, which disrupt the intestinal epithelial barrier by impairing TJ proteins. This impairment contributes to the development of gut dysbiosis, characterized by a compromised barrier integrity known as “leaky gut.” The resulting increased permeability allows pathogenic microbes and toxins from the gut lumen to enter the bloodstream, leading to endotoxemia marked by elevated LPS levels from gram-negative bacteria. This breach triggers an inflammatory response, upregulating pro-inflammatory cytokines and sustaining a chronic low-grade inflammatory environment. Inflammation and endotoxemia are associated with altered gut microbiota diversity and composition, promoting conditions like weight gain, obesity, metabolic syndrome, and an increased risk of obesity-related cancers. Abbreviations: CGN: Carrageenan; CMC: Carboxymethylcellulose; LPS: Lipopolysaccharides; P-80: Polysorbate-80; TJ: Tight junction; UPFs: Ultra-processed foods. Created in BioRender. Kounatidis, D. (2024) https://BioRender.com/g05j455. Assessed on 14 December 2024

This growing body of evidence underlines the importance of further investigating the relationship between UPFs, gut microbiota alterations, and the potential role of these factors in early-onset and obesity-associated cancers. Figure 3 illustrates the pathway from UPF overconsumption to obesity-associated cancer, highlighting gut dysbiosis as a potential key mechanism.

### Linking Ultra-Processed Food Overconsumption to Obesity-Related Cancer: Utilizing Current Evidence for Further Research

The modern lifestyle has driven a significant increase in the consumption of UPFs. Time constraints, demanding work schedules, and the convenience of ready-to-eat foods contribute to the rising demand for these options in daily life [141–144]. The ease of consumption and high palatability of UPFs often result in unintended overconsumption, which disrupts neurological and digestive functions and dysregulates hunger and satiety cues. These foods are generally low in essential nutrients, such as fiber, micronutrients, and bioactive compounds, while being high in sodium, trans fats, and refined sugars. This imbalance reduces satiety, increases the glycemic response, and disrupts insulin signaling, ultimately leading to persistent cravings, increased energy intake, and a self-reinforcing cycle of weight gain and obesity [141–144].

As illustrated, high UPF intake is associated with adverse metabolic outcomes, including elevated blood pressure and cardiovascular risks [145]. The excessive salt content of many UPFs significantly contributes to hypertension risk, while added simple sugars not only alter fructose metabolism but also intensify low-grade inflammation by promoting insulin resistance [145]. In response, pancreatic β-cells overproduce insulin to counteract insulin resistance, leading to cellular stress, structural damage, and impaired pancreatic function in a negative feedback loop [146]. Moreover, the high levels of trans and saturated fatty acids in UPFs disrupt lipid metabolism, hinder the breakdown of very-low-density lipoprotein cholesterol (VLDL-C), raise blood triglycerides, and reduce high-density lipoprotein (HDL) cholesterol levels [147].

The presence of chemical additives, preservatives, and synthetic antioxidants in UPFs further exacerbates health risks [148]. Harmful endocrine-disrupting chemicals, such as BPA, often leach from UPF packaging, contributing to metabolic and hormonal disturbances [49, 149]. Current evidence suggests that the obesogenic effects of UPFs, combined with exposure to potentially carcinogenic additives and pollutants from industrial processing, may elevate cancer risk. Notably, UPF consumption has been linked to disruptions in gut microbiota diversity and composition, as well as to impairment of the intestinal epithelial barrier, both of which are associated with an increased risk of obesity-related cancers [150, 151].

The growing body of evidence points to a significant link between ultra-processed food overconsumption and obesity-related cancer risks. The high levels of added sugars, unhealthy fats, and chemical additives in UPFs contribute to metabolic dysfunctions, endocrine disruption, and gut microbiome imbalances, all of which are known to drive obesity and inflammation [150–154]. These findings support the need for further research to clarify the mechanisms through which UPFs influence cancer pathways, as well as to inform dietary guidelines aimed at mitigating these health risks.

## Key References

- Lane MM, Davis JA, Beattie S, et al. Ultraprocessed food and chronic noncommunicable diseases: A systematic review and meta-analysis of 43 observational studies. Obes Rev. 2021;22(3):e13146. 10.1111/obr.13146.
  o This systematic review and meta-analysis investigated the association between consumption of ultra processed food and noncommunicable disease risk, morbidity and mortality.
- Chang K, Gunter MJ, Rauber F, et al. Ultra-processed food consumption, cancer risk and cancer mortality: a large-scale prospective analysis within the UK Biobank. EClinicalMedicine. 2023;56:101,840. 10.1016/j.eclinm.2023.101840.
  o This very recent manuscript focused on the significance associations between UPF consumption and risk of cancer and associated mortality for 34 site-specific cancers in a large cohort of British adults.
- Isaksen IM, Dankel SN. Ultra-processed food consumption and cancer risk: A systematic review and meta-analysis. Clin Nutr. 2023;42(6):919–928. 10.1016/j.clnu.2023.03.018.
  o This systematic review and meta-analysis explored the association between the consumption of UPF and cancer risk.
- Lane MM, Gamage E, Du S, et al. Ultra-processed food exposure and adverse health outcomes: umbrella review of epidemiological meta-analyses. BMJ. 2024;384:e077310. 10.1136/bmj-2023-077310.
  o This significant umbrella review and meta-analysis investigated associations between exposure to ultra-processed foods, as defined by the Nova food classification system, and adverse health outcomes.
- Barbaresko J, Bröder J, Conrad J, et al. Ultra-processed food consumption and human health: an umbrella review of systematic reviews with meta-analyses. Crit Rev Food Sci Nutr. 2024;1–9. 10.1080/10408398.2024.2317877.
  o This umbrella review and meta-analysis of the existing evidence of ultra-processed food consumption on human health.
- Moradi S, Entezari MH, Mohammadi H, et al. Ultra-processed food consumption and adult obesity risk: a systematic review and dose–response meta-analysis. Crit Rev Food Sci Nutr. 2023;63(2):249–260. 10.1080/10408398.2021.1946005.
  o A significant systematic review of the association between ultra-processed food consumption and the risk of overweight, obesity, and abdominal obesity in the general population.
- Dalamaga M, Kounatidis D, Tsilingiris D, et al. The Role of Endocrine Disruptors Bisphenols and Phthalates in Obesity: Current Evidence, Perspectives and Controversies. Int J Mol Sci. 2024;25(1):675. 10.3390/ijms25010675.
  o A review article elaborating the role of obesogenic EDCs, specifically BPA and phthalate plasticizers, in the development of obesity, encompassing in vitro, animal and epidemiologic studies.
- Petrelli F, Cortellini A, Indini A, et al. Association of Obesity With Survival Outcomes in Patients With Cancer: A Systematic Review and Meta-analysis. JAMA Netw Open. 2021;4(3):e213520. 10.1001/jamanetworkopen.2021.3520.
  o This systematic review and meta-analysis investigated the association between obesity and outcomes after a diagnosis of cancer.
- Bhaskaran K, Douglas I, Forbes H, et al. Body-mass index and risk of 22 specific cancers: a population-based cohort study of 5·24 million UK adults. Lancet. 2014;384(9945):755–765. 10.1016/S0140-6736(14)60892-8.
  o This cohort study evaluated the links between BMI and the most common site-specific cancers.
- Ryan AM, Duong M, Healy L, Ryan SA, et al. Obesity, metabolic syndrome and esophageal adenocarcinoma: epidemiology, etiology and new targets. Cancer Epidemiol. 2011;35(4):309–19. 10.1016/j.canep.2011.03.001.
  o A review article detailed how obesity and metabolic syndrome related to esophageal adenocarcinoma.
- Singh S, Olayinka OT, Fr J, et al. Food Additives’ Impact on Gut Microbiota and Metabolic Syndrome: A Systematic Review. Cureus. 2024;16(8):e66822. 10.7759/cureus.66822.
  o A review article detailed how dietary choices related to GM dysbiosis may contribute to metabolic syndrome.
- Wu M, Tian C, Zou Z, et al. Gastrointestinal Microbiota in Gastric Cancer: Potential Mechanisms and Clinical Applications-A Literature Review. Cancers (Basel). 2024;16(20):3547. 10.3390/cancers16203547.
  o A literature review article about novel therapeutic strategies targeting the microbiome, such as dietary interventions, probiotic and symbiotic supplementation, and fecal microbiota transplantation, are showing promise in cancer treatment.

## Data Availability

Not applicable (narrative review article)

## Abbreviations

BMI: Body Mass Index
BPA: Bisphenol-A
CI: Confidence Interval
CKD: Chronic Kidney Disease
CMC: Carboxymethylcellulose
CUP: Continuous Update Project
CVD: Cardiovascular Disease
DEHP: Di(ethylhexyl) Phthalate
E171: Titanium Dioxide
EOCRC: Early-Onset Colorectal Cancer
H.pylori: Helicobacter Pylori
HDL: High-Density Lipoprotein
HR: Hazard Ratio
IARC: International Agency for Research on Cancer
IGF-1: Insulin-Like Growth Factor-I
IL-6: Interleukin-6
LPS: Lipopolysaccharide
MEHP: Mono-2-Ethylhexyl Phthalate
OR: Odds Ratio
P-80: Polysorbate-80
RR: Relative Risk
SRR: Summary Relative Risk
T2DM: Type 2 Diabetes Mellitus
TJ: Tight Junction
TNF: α Tumor Necrosis Factor-Alpha
UPFs: Ultra-Processed Foods
VLDL: Very-Low-Density Lipoprotein
WHO: World Health Organization
ZO-1: Zona Occludens-1
↑: Increase
↓: Decrease
